# Relation of poverty with treatment-seeking behavior and antibiotic misuse among UTI patients in Pakistan

**DOI:** 10.3389/fpubh.2024.1357107

**Published:** 2024-03-15

**Authors:** Iltaf Hussain, Sundus Shukar, Muhammad Subhan Arshad, Muhammad Fawad Rasool, Jie Chang, Yu Fang

**Affiliations:** ^1^Department of Pharmacy Administration and Clinical Pharmacy, School of Pharmacy, Xi’an Jiaotong University, Xi’an, China; ^2^Center for Drug Safety and Policy Research, Xi’an Jiaotong University, Xi’an, China; ^3^Shaanxi Center for Health Reform and Development Research, Xi’an, China; ^4^Department of Pharmacy Practice, Faculty of Pharmacy, Bahauddin Zakariya University, Multan, Pakistan

**Keywords:** poverty, antibiotic misuse, UTI, treatment-seeking behavior, Pakistan

## Abstract

**Objective:**

The current study aimed to assess the relation between multi-dimension poverty, treatment-seeking behavior, and antibiotic misuse among urinary tract infection (UTI) patients.

**Method:**

A cross-sectional approach was utilized to recruit patients who had a history of UTI in the previous month from two provinces of Pakistan. The treatment-seeking behavior and antibiotic misuse data were collected on a self-developed questionnaire, whereas the poverty data were collected on a modified multi-dimension poverty index (MPI). Descriptive statistics were applied to summarize the data. The logistic regression analysis was carried out to assess the association of multi-dimension poverty with patient treatment-seeking behavior and antibiotic misuse.

**Results:**

A total of 461 participants who had UTI symptoms in the previous month were recruited. Most of the participants in the severely deprived stage treated the UTI (*p* < 0.001); however, there was a high proportion of the participants who consulted with friends and family for UTI treatment (*p* < 0.001). The patients with deprivation status (deprived and severely deprived) were less associated with formal consultation. The poorer subgroups were less likely to practice antibiotic course completion.

**Conclusion:**

The current study highlighted that poverty plays an important role in antibiotic misuse. Poorer subgroups were associated with informal consultations and the incompletion of the antibiotic course. Further studies are needed to explore the potential role of poverty in treatment-seeking behavior and antibiotic misuse.

## 1 Introduction

The antibiotic discovery was a major breakthrough in fighting against infectious diseases. However, the increasing resistance globally poses a major threat to public health and makes infectious diseases hard to treat (1). It has been estimated that antimicrobial resistance (AMR) was associated with 4.95 million deaths in 2019 and was most prevalent in low- and middle-income countries (2). The overuse and misuse of antibiotics are the key players in AMR. Human antibiotic consumption increased by 60% between 2001 and 2015 (3). In addition, the misuse of antibiotics is the widely acknowledged driver accounting for AMR (4). The misuse includes non-adherence to the antibiotic treatment, self-medication, and getting antibiotics without a prescription (5).

Poverty, in multi-dimensional terms, may be a significant factor for antibiotic misuse and, thus, for AMR. The healthcare system in developing countries is underfunded, and antibiotic dispensing is poorly regulated, thus promoting self-medication (6–8). There are limited studies available on the association between poverty and antibiotic misuse; however, there is a contradiction. A recent study reported that antibiotic misuse was more common among the least deprived and lowest among those living in severe multi-dimensional poverty (5). In contrast, one study suggested a more dominant role for poverty in antibiotic misuse and considered it a driver of AMR (9). A study from developing countries suggested that non-adherence to antibiotic treatment and self-medication were significantly associated with low education and poverty (10). On the other side, it was reported that people with more purchasing power could have more opportunities to self-medicate (6).

Pakistan is a developing country and the fifth most populous country in the world, with a poverty ratio of 39.8% (11, 12). On the other hand, in terms of antibiotic consumption, it is the third-highest country among low- and middle-income countries (3). Antibiotic resistance is increasing rapidly along with antibiotic consumption (13). The defined daily doses (DDDs) of antibiotics increased by 65% between 2000 and 2015 (3). The increasing consumption of antibiotics might contribute to the increasing resistance to antibiotics. In Pakistan, UTI is the most commonly occurring bacterial infection, with a prevalence of 16.1% (13). The resistance of first- and second-line antibiotics for treating UTI is prevailing in Pakistan (14). From these facts, we assumed that poverty may have a role in antibiotic misuse and self-medication, and UTI was used as a lens to better understand the role of multi-dimension poverty in treatment-seeking behavior, and antibiotic misuse in patients with a history of urinary tract infection (UTI).

## 2 Method

### 2.1 Study design, participants, and setting

The current study utilized a cross-sectional approach. The participants were recruited from two provinces of Pakistan (Khyber Pakhtunkhwa (KPK) and Punjab). The two provinces, among four provinces, were selected based on the high prevalence of UTI. The reported prevalence of UTI in Punjab and KPK was 74.5–81.5% (15–17) and 21.5–65.1% (14, 18–21), respectively. From each province, three districts were selected with the highest population (Supplementary Figure S1), as given by the Pakistan Bureau of Statistics (22). In each district, pharmacies or drug stores were selected through convenient sampling. The participants attending the selected pharmacies were asked about the symptoms, and those who had UTI symptoms in the previous month were selected for the study. The participants were conveniently selected for a duration of 6 months. In addition, the patients aged ≥18 years and living either in KPK or Punjab were recruited. This study was reported according to STROBE guidelines (23).

### 2.2 Study instrument and data collection

The questionnaire was developed based on the previous literature (5, 24–27). The initial draft of the questionnaire was face-validated by two experts with the same background. The suggestion given by the expert was adopted in the final version of the questionnaire. In addition, the questionnaire was also translated into Urdu by two experts in the field through forward and backward translation. The purpose of the translation was to provide convenience in understanding the questionnaire fror those who had problems reading and understanding English.

The questionnaire was composed of four domains. The first domain was related to the demographic characteristics of the participants. The second and third domains assess the participant’s behavior regarding treatment seeking and antibiotic use. The fourth domain is related to the reason for antibiotic misuse (Supplementary File: Appendix I).

Multi-dimension poverty was assessed using an adapted version of the multi-dimensional poverty index (MPI) for developing countries (5, 28). The MPI measured poverty using three dimensions, including education, health, and living standards (Supplementary File: Appendix II). The poverty was classified into four categories using counting methodology, as previously reported (5) (less than 20%, not deprived; 20% to less than 34%, vulnerable to poverty; 34–49%, deprived; and more than 50%, living in severe poverty).

The data were collected using a self-developed questionnaire. The local data collector was utilized for data collection (*n* = 2 from each province). The data collector was trained regarding the study objectives and inclusion and exclusion criteria for the recruitment of participants to the study. The participants were randomly selected from pharmacies or drug stores in the selected districts by the data collectors. The data were collected using Google form (Google LLC, Inc.). All the collected data were imported into Microsoft Excel to screen for errors.

### 2.3 Study ethics

The study was approved by the Health Science Center, Xi’an Jiaotong University, Xi’an, China. Patient confidentiality was maintained throughout the study. Informed consent was obtained from each participant. Those who voluntarily agreed to participate were recruited for the current study.

### 2.4 Statistical analysis

Descriptive statistics were applied to summarize the data. The categorical variable was presented as frequency and percentages. The chi-square and Fisher’s exact tests (if the cell count is less than 5) were used to assess the association of the study variables with poverty. A logistic regression analysis was used to assess the association of multi-dimension poverty with treatment-seeking behavior and antibiotic misuse. Model 1 measured the association of multi-dimension poverty with treatment-seeking behavior and antibiotic misuse, adjusted for age and gender. In model 2, we also added working status. The adjusted odds ratios (aORs) with a 95% confidence interval were reported for the logistic regression analysis. The consultation was grouped into formal (physician and pharmacist) and informal (friends and family and others) consultation in the logistic regression analysis. The *p*-value was considered significant throughout the analysis if it was ≤0.05.

## 3 Results

A total of 461 participants were recruited for the current study. The frequency of UTI was 19.1%, as shown in Supplementary Figure S2. Most participants fell in the age range of 18–30 years (55.2%) and were female (54.5%). Most of the participants had an undergraduate level of education (34.4%), followed by primary education (26.6%). The majority of the participants were shown unemployment and working status (75.3%). The detailed demographic characteristics can be seen in Table 1.

**Table 1:** Demographic characteristics of the participants (*n* = 462).

	Overall N (%)	Province	
		Kpk N (%)	Punjab N (%)
	*N* = 462	*N* = 216	*N* = 246
Age (yrs.)			
18–30	255 (55.2)	123 (57.2)	131 (53.3)
31–40	21 (4.5)	6 (2.8)	15 (6.1)
41–50	78 (16.9)	39 (18.1)	39 (15.9)
51–60	75 (16.2)	32 (14.9)	43 (17.5)
>60	33 (7.1)	15 (7.0)	18 (7.3)
Gender			
Male	210 (45.5)	102 (47.4)	107 (43.5)
Female	252 (54.5)	113 (52.6)	139 (56.5)
Education			
No education	27 (5.8)	8 (3.7)	19 (7.7)
Primary education	123 (26.6)	57 (26.5)	66 (26.8)
Middle and secondary education	66 (14.3)	31 (14.4)	35 (14.2)
Undergraduate	159 (34.4)	76 (35.3)	82 (33.3)
Postgraduate	87 (18.8)	43 (20.0)	44 (17.9)
Working status			
Not employed	348 (75.3)	165 (76.7)	183 (74.4)
Employed	114 (24.7)	50 (23.3)	63 (25.6)

Kpk^*^, Khyber Pakhtunkhwa.

In the current study, most of the population was in vulnerable (41.1%) and severe deprivation (40.9%) poverty status. Most of the population treated the UTI (61.7%); however, less than half consulted with their friends and family for the treatment of the UTI (44.2%). Among those who treated the UTI, 81.4% used antibiotics. More than half of the participants were practice self-medication with antibiotics (67.7%). Moreover, 51.6% of the participants did not complete the antibiotic course, and 28.3% of the participants skipped the antibiotic dose. Most of the participants get the antibiotic without a prescription because they do not have a prescription (71.4%). The details can be seen in Table 2.

**Table 2:** Distribution of study variables across two provinces (*n* = 462).

	Overall N (%)	Province		*p*-value^a^
		Kpk N (%)	Punjab N (%)	
Multi-dimension poverty				
Not deprived	33 (7.1)	17 (7.9)	16 (6.5)	0.73
Vulnerable	190 (41.1)	92 (42.8)	97 (39.4)	
Deprived	50 (10.8)	21 (9.8)	29 (11.8)	
Severely deprived	189 (40.9)	85 (39.5)	104 (42.3)	
UTI Treated				
No	177 (38.3)	82 (38.1)	95 (38.6)	0.91
Yes	285 (61.7)	133 (61.9)	151 (61.4)	
Consultation				
Physician	75 (26.3)	32 (24.1)	43 (28.5)	0.58*
Pharmacist	78 (27.4)	41 (30.8)	36 (23.8)	
Friends and Family	126 (44.2)	57 (42.9)	69 (45.7)	
Other	6 (2.1)	3 (2.3)	3 (2.0)	
Use of antibiotics for treatment of UTI				
No	65 (18.6)	32 (19.4)	33 (17.9)	0.72
Yes	285 (81.4)	133 (80.6)	151 (82.1)	
Antibiotics used				
Ciprofloxacin	211 (75.1)	96 (73.8)	114 (76.0)	0.90
Levofloxacin	55 (19.6)	27 (20.8)	28 (18.7)	
Other	15 (5.3)	7 (5.4)	8 (5.3)	
Self-medication				
No	90 (32.3)	38 (29.7)	51 (34.0)	0.44
Yes	189 (67.7)	90 (70.3)	99 (66.0)	
Antibiotic course completed				
No	144 (51.6)	67 (52.3)	76 (50.7)	0.78
Yes	135 (48.4)	61 (47.7)	74 (49.3)	
Skip antibiotic dose				
No	99 (71.7)	45 (72.6)	54 (71.1)	0.84
Yes	39 (28.3)	17 (27.4)	22 (28.9)	

Kpk, Khyber Pakhtunkhwa; ^a^, chi-square test; *, Fisher’s exact test; UTI, urinary tract infection.

The total of some variables is equal to 285, presenting data only from those who treat the UTI.

Most of the participants in the severely deprived stage, treated the UTI (*p* < 0.001); however, there was a high proportion of the participants who consulted with friends and family for UTI treatment (*p* < 0.001). Most of the population in the severely deprived stage practiced self-medication (*p* < 0.001) and incompletion of the antibiotic course (*p* < 0.001) as shown in Table 3. There was no significant variation across the provinces in terms of treatment-seeking behavior and antibiotic misuse, as shown in Supplementary Table S2.

**Table 3:** Association of the treatment-seeking behavior and antibiotic misuse across poverty dimensions (*n* = 462).

	Multi-dimension poverty				*p*-value^a^
	Not deprived	Vulnerable	Deprived	Severely deprived	
UTI Treated					
No	15 (45.5)	83 (43.7)	28 (56.0)	51 (27.0)	<0.001
Yes	18 (54.5)	107 (56.3)	22 (44.0)	138 (73.0)	
Consultation					
Physician	6 (33.3)	54 (50.5)	9 (40.9)	6 (4.3)	<0.001*
Pharmacist	9 (50.0)	42 (39.3)	6 (27.3)	21 (15.2)	
Friends and Family	3 (16.7)	9 (8.4)	6 (27.3)	108 (78.3)	
Other	0 (0.0)	2 (1.9)	1 (4.5)	3 (2.2)	
Use of antibiotics for treatment of UTI					
No	5 (21.7)	32 (23.0)	7 (24.1)	21 (13.2)	0.13
Yes	18 (78.3)	107 (77.0)	22 (75.9)	138 (86.8)	
Self-medication					
No	6 (33.3)	59 (55.1)	10 (45.5)	15 (11.4)	<0.001
Yes	12 (66.7)	48 (44.9)	12 (54.5)	117 (88.6)	
Antibiotic course completed					
No	1 (5.6)	21 (19.6)	7 (31.8)	115 (87.1)	<0.001
Yes	17 (94.4)	86 (80.4)	15 (68.2)	17 (12.9)	
Skip antibiotic dose					
No	12 (66.7)	64 (73.6)	11 (73.3)	12 (66.7)	0.89*
Yes	6 (33.3)	23 (26.4)	4 (26.7)	6 (33.3)	

Kpk, Khyber Pakhtunkhwa; ^a^, chi-square test; *, Fisher’s exact test; UTI, urinary tract infection.

The total of some variables is equal to 285, presenting data only from those who treat the UTI.

Regarding treatment-seeking behavior, the patients aged >30 years were more likely to treat the UTI and use antibiotics compared to those aged ≤30 years. Being male was more associated with UTI treatment, seeking formal consultation, and the use of antibiotics for the treatment of UTI. Patients with deprived and severely deprived status were less likely to be associated with formal consultation. The details can be seen in Table 4.

**Table 4:** Logistic regression results showing the association of multi-dimension poverty with patient treatment-seeking behavior (*n* = 462).

	Model 1 (age + gender)			Model 2 (Model 1 + working status)		
	UTI treated	Formal consultation	antibiotic used	UTI treated	Formal consultation	antibiotic used
	aOR (95% CI)	aOR (95% CI)	aOR (95% CI)	aOR (95% CI)	aOR (95% CI)	aOR (95% CI)
Poverty status						
SD	**1.04 (0.36–3.01)**	0.82 (0.27–2.42)	0.95 (0.34–2.69)	**1.22 (0.42–3.56)**	**0.95 (0.32–2.84)**	**1.16 (0.40–3.34)**
D	0.58 (0.32–1.68)	**0.72 (0.24–2.15)**	0.41 (0.14–1.18)	**0.64 (0.22–1.85)**	**0.78 (0.26–2.32)**	0.45 (0.15–1.32)
VD	**1.04 (0.49–2.19)**	**1.3 (0.62–2.75)**	0.761 (0.36–1.61)	1.01 (0.47–2.16)	**1.28 (0.60–2.72)**	**0.73 (0.34–1.56)**
ND	Ref.	Ref.	Ref.	Ref.	Ref.	Ref.
Age (yrs.)						
>60	**3.87 (1.82–5.42)**	0.64 (0.35–1.80)	7.56 (1.78–31.99)	**3.79 (1.80–5.26)**	**0.63 (0.34–1.76)**	**7.38 (1.74–31.27)**
51–60	2.57 (0.99–6.67)	**0.28 (0.09–0.86)**	2.03 (0.8–5.13)	**2.42 (0.93–6.31)**	**0.26 (0.09–0.80)**	1.86 (0.73–4.74)
41–50	4.10 (1.72–9.91)	**0.35 (0.13–0.92)**	**3.32 (1.42–7.77)**	**3.63 (1.49–8.82)**	**0.30 (0.11–0.80)**	**2.85 (1.21–6.74)**
31–40	3.06 (1.04–9.01)	0.62 (0.21–1.8)	**1.83 (0.67–4.99)**	2.44 (0.81–7.33)	**0.51 (0.18–1.50)**	**1.42 (0.51–3.91)**
18–30	Ref.	Ref.	Ref.	Ref.	Ref.	Ref.
Gender						
Male	**1.76 (1.09–2.82)**	1.62 (1.02–2.63)	**1.44 (0.9–2.3)**	**1.43 (0.87–2.34)**	**1.37 (0.82–2.26)**	1.14 (0.70–1.86)
Female	Ref.	Ref.	Ref.	Ref.	Ref.	Ref.
Working status						
Employed				**2.16 (1.29–3.61)**	**1.84 (1.12–3.03)**	2.41 (1.46–3.99)
Not employed				Ref.	Ref.	Ref.

SD, severely deprived; D, deprived; VD, vulnerable to deprivation; ND, Not deprived; aOR, adjusted odds ratio; CI, confidence interval; SMA, self-medication with antibiotics; Ref., reference; bold - *p* < 0.05.

To assess the association of multi-dimension poverty with treatment-seeking behavior: Model 1 was adjusted for age and gender (as confounding variables), and in Model 2, working status was also incorporated as working status may act as a confounding variable.

Patients aged greater than 30 years were more likely than those aged equal to or less than 30 years to practice self-medication and skip the dose. Male participants were more likely to practice self-medication, complete the course completion and skip the dose than female participants. Moreover, patients in the deprivation stage compared to patients in the not-deprived stage were less likely to complete the course and practice self-medication, as shown in Table 5.

**Table 5:** Logistic regression results showing the association of multi-dimension poverty with antibiotic misuse (*n* = 462).

	Model 1 (age + gender)			Model 2 (Model 1 + working status)		
	SMA	Course completion	Skip dose	SMA	Course completion	Skip dose
	aOR (95% CI)	aOR (95% CI)	aOR (95% CI)	aOR (95% CI)	aOR (95% CI)	aOR (95% CI)
Poverty status						
SD	0.37 (0.11–1.18)	**0.49 (0.16–1.47)**	0.86 (0.25–3.02)	0.44 (0.14–1.41)	**0.59 (0.19–1.81)**	**0.89 (0.25–3.16)**
D	0.34 (0.10–1.14)	0.48 (0.16–1.43)	**0.92 (0.25–3.37)**	0.37 (0.11–1.28)	0.52 (0.17–1.55)	0.94 (0.26–3.45)
VD	0.53 (0.24–1.17)	0.75 (0.36–1.57)	0.94 (0.36–2.46)	0.51 (0.23–1.13)	0.71 (0.33–1.52)	0.94 (0.36–2.45)
ND	Ref.	Ref.	Ref.	Ref.	Ref.	Ref.
Age (yrs.)						
>60	4.94 (2.23–7.14)	**0.30 (0.07–1.33)**	3.93 (1.56–4.35)	4.62 (2.14–6.85)	**0.29 (0.07–1.30)**	**2.83 (1.42–3.65)**
51–60	1.31 (0.86–1.59)	1.02 (0.08–1.05)	**1.25 (0.87–1.58)**	1.21 (0.79–1.43)	0.25 (0.07–0.96)	**1.17 (0.76–1.47)**
41–50	1.15 (0.41–2.08)	0.27 (0.10–0.76)	1.06 (0.72–1.54)	**1.01 (0.37–1.58)**	**0.22 (0.08–0.61)**	1.01 (0.63–1.47)
31–40	1.12 (0.37–1.27)	0.69 (0.24–2.00)	**0.98 (0.63–1.36)**	**1.08 (0.29–1.17)**	**0.52 (0.17–1.52)**	0.92 (0.54–1.29)
18–30	Ref.	Ref.	Ref.	Ref.	Ref.	Ref.
Gender						
Male	**1.88 (1.08–3.27)**	1.48 (0.91–2.41)	1.15 (0.66–1.98)	**1.61 (0.90–2.87)**	**1.13 (0.67–1.90)**	**1.12 (0.64–1.96)**
Female	Ref.	Ref.	Ref.	Ref.	Ref.	Ref.
Working status						
Employed				**1.89 (1.11–3.22)**	**2.42 (1.45–4.06)**	**1.12 (0.64–1.96)**
Not employed				Ref.	Ref.	Ref.

SD, severely deprived; D, deprived; VD, vulnerable to deprivation; ND, Not deprived; aOR, adjusted odds ratio; CI, confidence interval; SMA, self-medication with antibiotics; Ref., reference; bold - *p* < 0.05.

To assess the association of multi-dimension poverty with antibiotic misuse: Model 1 was adjusted for age and gender (as confounding variables), and in Model 2, working status was also incorporated as working status may act as a confounding variable.

**Figure 1:**
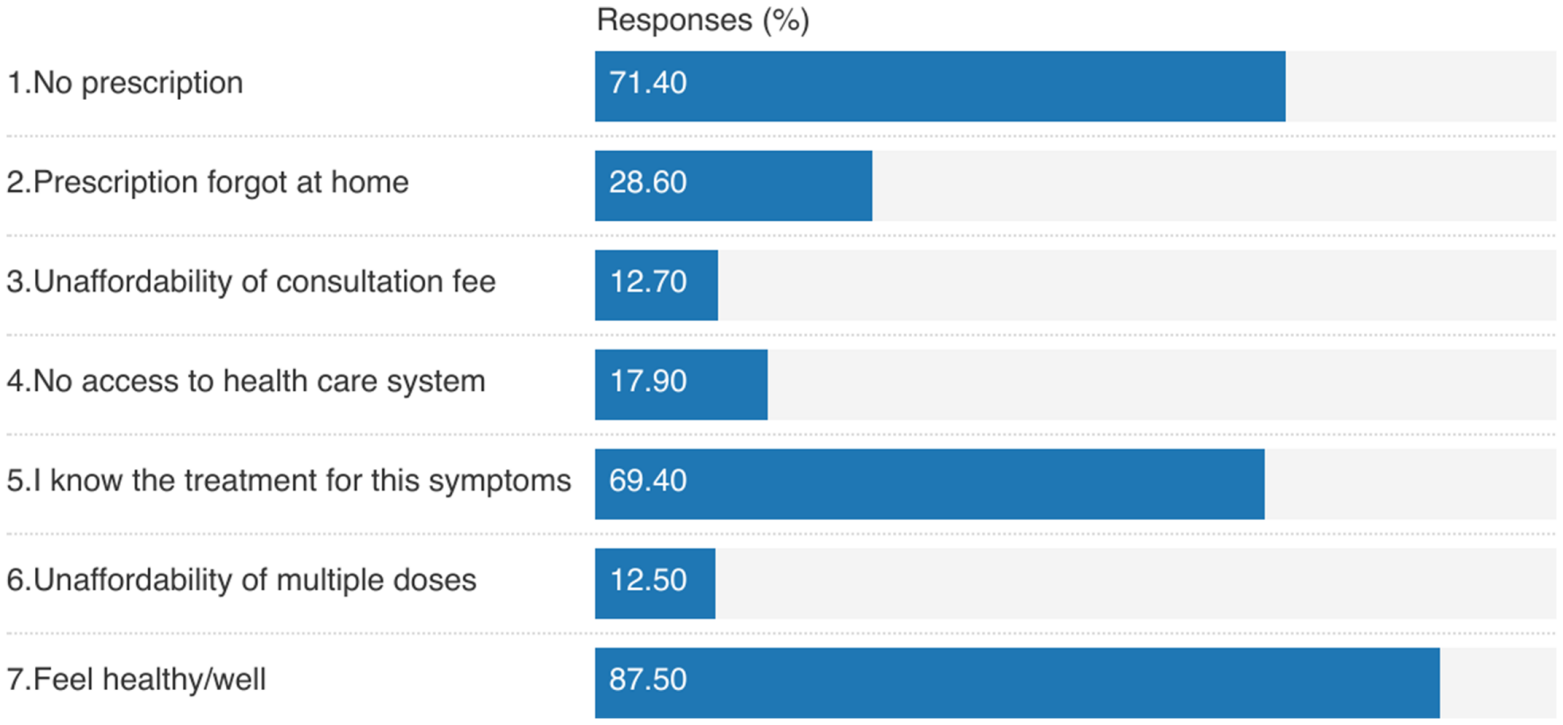
Reasons for: (1) getting antibiotics without prescription (1, 2), (2) not having prescription (3–5), and (3) not completing the antibiotic course (6, 7).

Familiarity with UTI symptoms (69.4%) was the most reported reason for not having a prescription. Moreover, feeling healthy was the most common reason for not completing an antibiotic course (87.5%), as shown in Figure 1.

## 4 Discussion

To the best of our knowledge, this is the first study from Pakistan that assesses the role of poverty in treatment-seeking behavior and antibiotic misuse among UTI patients. The current study highlighted that most of the patients with severely deprived poverty status had treated the UTI, but less than half of the participants practiced informal consultation (friends and family) for their treatment. Antibiotic self-medication was less practiced in the deprived subgroups. Poorer subgroups (vulnerable to deprivation, deprived, and severely deprived) were significantly associated with the incompletion of the antibiotic course. The most reported reason for self-medication was not having a prescription and familiarity with the treatment of UTI. Feeling healthy was the most common reason for not completing the antibiotic course.

Self-medication with antibiotics is globally prevalent and significantly contributes to the prevalence of AMR (29, 30). In the current study, the deprived subgroups were less likely to practice formal consultation (pharmacist and physician). The reasons may be familiarity and previous experience with the disease. As reported in the current study, 69.4% of the respondents have used antibiotics without a prescription because they knew the treatment for these symptoms. In addition, the difficulty in managing healthcare costs may be an obstacle to formal consultation and optimal use of antibiotics. The unaffordability of the treatment cost promotes self-medication. It has been reported previously that the high cost of consulting a physician (31), lack of access to medical care (32), lack of confidence in physicians (31), lack of health insurance (33), and easy access to antibiotics (31, 34) also have a direct association with the promotion of self-medication or an indirect relation in promoting patient behavior toward self-medication. However, in the current study, the poor subgroups were less likely to practice self-medication with antibiotics as compared to the not-deprived subgroup. It may be because the poor subgroup can not afford the antibiotic cost. The inability to cover the cost of antibiotics may be the reason for not practicing self-medication with antibiotics in the current study. It has been reported previously that the inability to cover transport and drug costs may lead to informal consultation and traditional treatment options.

The in-completion of the antibiotics course is the patient-related behavior associated with antibiotic misuse. This is one of the significant contributors to AMR by partially exposing bacteria to antibiotics. The un-killed exposed bacteria become resistant by mutation (35, 36). In line with previously reported studies (6, 10), we found that non-adherence (course incompletion) was more common among patients living in the most deprived conditions. However, a recent study from developing countries showed that non-adherence was more common in populations not living in multi-dimension poverty (5). However, patient behavior may play a role in this non-adherence, as most of the participants in the current study reported feeling healthy as a reason for the incompletion of the antibiotic course. In addition, the lack of access to the healthcare system and the inability to meet the treatment cost may also play a role in the non-adherence to the antibiotic course (5). Therefore, it is suggested that patient-related behavior be incorporated in to the design of the policies. In addition, awareness regarding antibiotic use and duration of use should be given to people through educational seminars, consultation with physicians, and receiving antibiotics from pharmacists.

The current study has several limitations. First, we conveniently selected patients from the community pharmacies and drug stores in the three districts in each province. Therefore, the results of this study may not be generalizable to the whole population. Second, recall bias is one of the major limitations of the questionnaire-based surveys. To minimize this, we limit the patients to a 1-month recall. Third, the patients were not categorized into subgroups based on severity, which may act as a confounding factor in the current study. Fourth, the patient population was limited to the community, which is not representative of the patient population attending hospitals or clinics. Finally, our outcomes were self-reported, and we cannot rule out the reporting bias.

## 5 Conclusion

The current study highlighted that poverty plays an important role in antibiotic misuse. Poorer subgroups were associated with informal consultations and the incompletion of the antibiotic course. The current study, along with patient behavior, also highlighted easy access to antibiotics. Therefore, it is recommended that policies regarding access to antibiotics be implemented effectively and health insurance be given to afford the treatment expenditure. In addition, regulatory bodies should emphasize patient education regarding antibiotic use. Further studies are needed to explore the potential role of poverty in antibiotic misuse by incorporating individual and contextual factors such as sociodemographics, cultural, and pluralistic healthcare systems, and regulatory infrastructure.

## Data availability statement

The original contributions presented in the study are included in the article/Supplementary material, further inquiries can be directed to the corresponding authors.

## Ethics statement

The studies involving humans were approved by Health Science Center, Xi’an Jiaotong University, Xi’an China. The studies were conducted in accordance with the local legislation and institutional requirements. The participants provided their written informed consent to participate in this study.

## Author contributions

IH: Conceptualization, Formal analysis, Methodology, Project administration, Writing – original draft, Writing – review & editing. SS: Conceptualization, Methodology, Project administration, Writing – original draft, Writing – review & editing. MS: Formal analysis, Writing – original draft, Writing – review & editing. MR: Data curation, Formal analysis, Writing – original draft, Writing – review & editing. JC: Conceptualization, Methodology, Project administration, Writing – original draft, Writing – review & editing. YF: Conceptualization, Formal analysis, Funding acquisition, Methodology, Project administration, Supervision, Writing – original draft, Writing – review & editing.

## References

[1] Uddin TM Chakraborty AJ Khusro A Zidan BRM Mitra S Emran TB. Antibiotic resistance in microbes: history, mechanisms, therapeutic strategies and future prospects. J Infect Public Health. (2021) 14:1750–66. doi: 10.1016/j.jiph.2021.10.020, PMID: 34756812

[2] Charani E McKee M Balasegaram M Mendelson M Singh S Holmes AH. Global burden of antimicrobial resistance: essential pieces of a global puzzle. Lancet. (2022) 399:2346–7. doi: 10.1016/S0140-6736(22)00935-7 35753334

[3] Klein EY Van Boeckel TP Martinez EM Pant S Gandra S Levin SA. Global increase and geographic convergence in antibiotic consumption between 2000 and 2015. Proc Natl Acad Sci USA. (2018) 115:E3463–70. doi: 10.1073/pnas.1717295115, PMID: 29581252 PMC5899442

[4] Subramaniam G Girish M. Antibiotic resistance -a cause for reemergence of infections. Indian J Pediatr. (2020) 87:937–44. doi: 10.1007/s12098-019-03180-3 32026301

[5] Green DL Keenan K Fredricks KJ Huque SI Mushi MF Kansiime C. The role of multidimensional poverty in antibiotic misuse: a mixed-methods study of self-medication and non-adherence in Kenya, Tanzania, and Uganda. Lancet Glob Health. (2023) 11:e59–68. doi: 10.1016/s2214-109x(22)00423-5, PMID: 36521953

[6] Torres NF Chibi B Middleton LE Solomon VP Mashamba-Thompson TP. Evidence of factors influencing self-medication with antibiotics in low and middle-income countries: a systematic scoping review. Public Health. (2019) 168:92–101. doi: 10.1016/j.puhe.2018.11.018, PMID: 30716570

[7] Do NTT Vu HTL Nguyen CTK Punpuing S Khan WA Gyapong M. Community-based antibiotic access and use in six low-income and middle-income countries: a mixed-method approach. Lancet Glob Health. (2021) 9:e610–9. doi: 10.1016/s2214-109x(21)00024-3, PMID: 33713630 PMC8050200

[8] Batista AD Rodrigues D Figueiras A Zapata-Cachafeiro M Roque F Herdeiro MT. Antibiotic dispensation without a prescription worldwide: a systematic review. Antibiotics. (2020) 9:786. doi: 10.3390/antibiotics9110786, PMID: 33171743 PMC7694985

[9] World Health Organization. Communicable diseases. Overcoming antimicrobial resistance. Geneva: World Health Organization (2000).

[10] Aslam A Gajdács M Zin CS Ab Rahman NS Ahmed SI Zafar MZ. Evidence of the practice of self-medication with antibiotics among the lay public in low- and middle-income countries: a scoping review. Antibiotics. (2020) 9:597. doi: 10.3390/antibiotics9090597, PMID: 32932630 PMC7558641

[11] United Nations Population Fund. (2022). World population dashboard-Pakistan. Available at:https://www.unfpa.org/data/world-population/PK.

[12] World Bank. World bank data-Pakistan. (2018). Available at: https://data.worldbank.org/topic/poverty?end=2018&locations=PK&start=1987.

[13] Bilal H Khan MN Rehman T Hameed MF Yang X. Antibiotic resistance in Pakistan: a systematic review of past decade. BMC Infect Dis. (2021) 21:244. doi: 10.1186/s12879-021-05906-1, PMID: 33676421 PMC7937258

[14] Bullens M de Cerqueira MA Raziq S Lee J Khalid GG Khan SN. Antibiotic resistance in patients with urinary tract infections in Pakistan. Public Health Action. (2022) 12:48–52. doi: 10.5588/pha.21.0071, PMID: 35317540 PMC8908872

[15] Sabir S Ahmad Anjum A Ijaz T Asad Ali M Ur Rehman Khan M Nawaz M. Isolation and antibiotic susceptibility of *E. coli* from urinary tract infections in a tertiary care hospital. Pak J Med Sci. (2014) 30:389–92. doi: 10.12669/pjms.302.4289 24772149 PMC3999016

[16] Hussain A Sohail M Abbas Z. Prevalence of *Enterococcus faecalis* mediated UTI and its current antimicrobial susceptibility pattern in Lahore, Pakistan. J Pak Med Assoc. (2016) 66:1232–6. PMID: 27686295

[17] Asmat U Mumtaz MZ Malik A. Rising prevalence of multidrug-resistant uropathogenic bacteria from urinary tract infections in pregnant women. J Taibah Univ Med Sci. (2021) 16:102–11. doi: 10.1016/j.jtumed.2020.10.010, PMID: 33603638 PMC7858016

[18] Khatoon I Khanam S Azam A Qadeer S Naz S Hassan NU. Incidence pattern, antibiotic susceptibility pattern and associated risk factors of bacterial Uropathogens among general population of Pakistan. Infect Drug Resist. (2023) 16:4995–5005. doi: 10.2147/idr.S418045, PMID: 37551281 PMC10404436

[19] Faiza A Sardar M Aftab Ahmad A Tehreem A Abdul Shaheed A Sobia N. Multi-drug resistance pattern of bacterial isolates from urinary tract infection. Pak J Pharm Sci. (2023) 36:1107–12. doi: 10.36721/PJPS.2023.36.4.REG.1107-1112.1 37599485

[20] Anis R Jahanzeb M Siddiqui TS Idris M. Frequency and clinical presentation of UTI among children of Hazara division, Pakistan. J Ayub Med Coll Abbottabad. (2008) 20:63–5. 19024189

[21] Ahmad S Ali F Qureshi SA Uzma B Shakeela Q Sabir MS. The evaluation of antibiotic susceptibility pattern and associated risk factors of UTI in tertiary care hospital of Peshawar. Pak J Pharm Sci. (2022) 35:897–903. doi: 10.36721/PJPS.2022.35.3.SP.897-903.1 35791585

[22] Pakistan Bureau of Statistics. 7th population and housing Census-2023 ‘the digital census’. (2023). Available at: https://www.pbs.gov.pk/content/announcement-results-7th-population-and-housing-census-2023-digital-census.

[23] von Elm E Altman DG Egger M Pocock SJ Gøtzsche PC Vandenbroucke JP. The strengthening the reporting of observational studies in epidemiology (STROBE) statement: guidelines for reporting observational studies. J Clin Epidemiol. (2008) 61:344–9. doi: 10.1016/j.jclinepi.2007.11.008 18313558

[24] McGurn A Watchmaker B Adam K Ni J Babinski P Friedman H. Socioeconomic status and determinants of pediatric antibiotic use. Clin Pediatr. (2021) 60:32–41. doi: 10.1177/0009922820941629, PMID: 32748648 PMC7983842

[25] Alsan M Kammili N Lakshmi J Xing A Khan A Rani M. Poverty and community-acquired antimicrobial resistance with extended-Spectrum β-lactamase-producing organisms, Hyderabad, India. Emerg Infect Dis. (2018) 24:1490–6. doi: 10.3201/eid2408.171030, PMID: 30014842 PMC6056104

[26] Planta MB. The role of poverty in antimicrobial resistance. J Am Board Fam Med. (2007) 20:533–9. doi: 10.3122/jabfm.2007.06.070019, PMID: 17954860

[27] Kliemann BS Levin AS Moura ML Boszczowski I Lewis JJ. Socioeconomic determinants of antibiotic consumption in the state of São Paulo, Brazil: the effect of restricting over-the-counter sales. PLoS One. (2016) 11:e0167885. doi: 10.1371/journal.pone.0167885, PMID: 27941993 PMC5152856

[28] Alkire S Santos ME. Measuring acute poverty in the developing world: robustness and scope of the multidimensional poverty index. World Dev. (2014) 59:251–74. doi: 10.1016/j.worlddev.2014.01.026

[29] Shehnaz SI Agarwal AK Khan N. A systematic review of self-medication practices among adolescents. J Adolesc Health. (2014) 55:467–83. doi: 10.1016/j.jadohealth.2014.07.001 25245937

[30] Ahmed I King R Akter S Akter R Aggarwal VR. Determinants of antibiotic self-medication: a systematic review and meta-analysis. Res Soc Adm Pharm. (2023) 19:1007–17. doi: 10.1016/j.sapharm.2023.03.009, PMID: 37019706

[31] Ramay BM Lambour P Cerón A. Comparing antibiotic self-medication in two socio-economic groups in Guatemala City: a descriptive cross-sectional study. BMC Pharmacol Toxicol. (2015) 16:11. doi: 10.1186/s40360-015-0011-3, PMID: 25928897 PMC4418049

[32] Rathish D Wickramasinghe ND. Prevalence, associated factors and reasons for antibiotic self-medication among dwellers in Anuradhapura: a community-based study. Int J Clin Pharm. (2020) 42:1139–44. doi: 10.1007/s11096-020-01065-6, PMID: 32458226

[33] Mohanna M. Self-medication with antibiotic in children in Sana'a City, Yemen. Oman Med J. (2010) 25:41–3. doi: 10.5001/omj.2010.10, PMID: 22125697 PMC3215380

[34] Mate I Come CE Gonçalves MP Cliff J Gudo ES. Knowledge, attitudes and practices regarding antibiotic use in Maputo City, Mozambique. PLoS One. (2019) 14:e0221452. doi: 10.1371/journal.pone.0221452, PMID: 31437215 PMC6705831

[35] Taha MK Deghmane AE. Evolution of resistance to antibiotics in *Neisseria meningitidis*: any reasons for concern? J Infect Dis. (2022) 225:1869–70. doi: 10.1093/infdis/jiac095, PMID: 35266521

[36] Płusa T Konieczny R Baranowska A Szymczak Z. The growing resistance of bacterial strains to antibiotics. Pol Merkur Lekarski. (2019) 25:106–10. 31557140

