# Supplementary material for: Relation of poverty with treatment-seeking behavior and antibiotic misuse among UTI patients in Pakistan
Source: Front Public Health. 2024 Mar 15;12:1357107. doi: 10.3389/fpubh.2024.1357107 (PMC10978578; doi:10.3389/fpubh.2024.1357107)
Supplement: Supplementary file 1 [file Data_Sheet_1.docx]

# **Supplementary materials**

## **Appendix I: Questionnaire to assess the participant’s treatment seeking behavior and antibiotic misuse**

1. Are you Agree to participate in the study?

Yes

No (end)

1. In previous one month, did you had this symptom (s) (i) burning pain while urinating and/or (ii) an urgent need to urinate and/or (iii) pain or pressure in the lower abdomen, and/or (iv) cloudy, dark, bloody or strange smelling urine and (v) these symptoms can also be associated with feelings of tiredness/illness or fever.

Yes

No (end)

1. **What is your age (year)?**

18-30

31-40

41-50

51-60

>60

1. **What is your gender?**

Male

Female

1. **What is your highest education level?**

No education

Primary level

Middle level

Undergraduate

Postgraduate

1. **What is your working status?**

Employed

Not employed

1. **To whom you consult regarding the treatment for the symptoms?**

Physician

Pharmacist

Family and friends

1. **Did you use antibiotics for the treatment of the symptoms?**

Yes

No (end)

1. **Which antibiotics you used for the treatment of the symptoms (Open ended)**
2. **How did you get the antibiotics from Pharmacy/drugstore?**

With prescription

Without prescription

1. **Did you complete the antibiotic course?**

Yes

No (Go to Q15)

1. **Did you skip the dose of antibiotic during the course of treatment?**

Yes

No

1. **What was the reason of getting antibiotics without prescription?**

I don’t have prescription (Go to Q14)

I have prescription but forgot at home

1. **What was the reason of not having prescription?**

I didn’t visit physician because I can’t afford the consultation fee,

I know the treatment for these symptoms,

I cannot access to the health care system (Hospital), other

1. **What was the reason of not completing the antibiotic course?**

I feel healthy/well,

I can’t afford the multiple doses of the antibiotics other

## **Appendix II: Supplementary Table S1. Multi-dimension poverty index (MPI)**

| **Dimension** | **Indicators** | **Deprivation cut-off** | **Weighing** |
| --- | --- | --- | --- |
| Education | 1) Level of education | If the patient has less than a secondary education | 1/3 |
| Health | 2) Disability | If the patient is disabled | 1/6 |
|  | 3) Chronic illness | If the patient has one of the following illnesses: diabetes, heart disease, asthma, hypertension/high blood pressure, HIV/AIDS, heart problems, stroke, and cancer. | 1/6 |
| Standard of living | 4) Sanitation | No access to private flush toilet | 1/12 |
|  | 5) Asset ownership | Does not own more than one of the following: TV, computer, radio, fridge, cellular phone (smart or not), fishing boat, motor | 1/12 |
|  | 6) Electricity | Does not have access to mains electricity | 1/12 |
|  | 7) Water | Do have access to the private protected water source | 1/12 |

## **Figures**


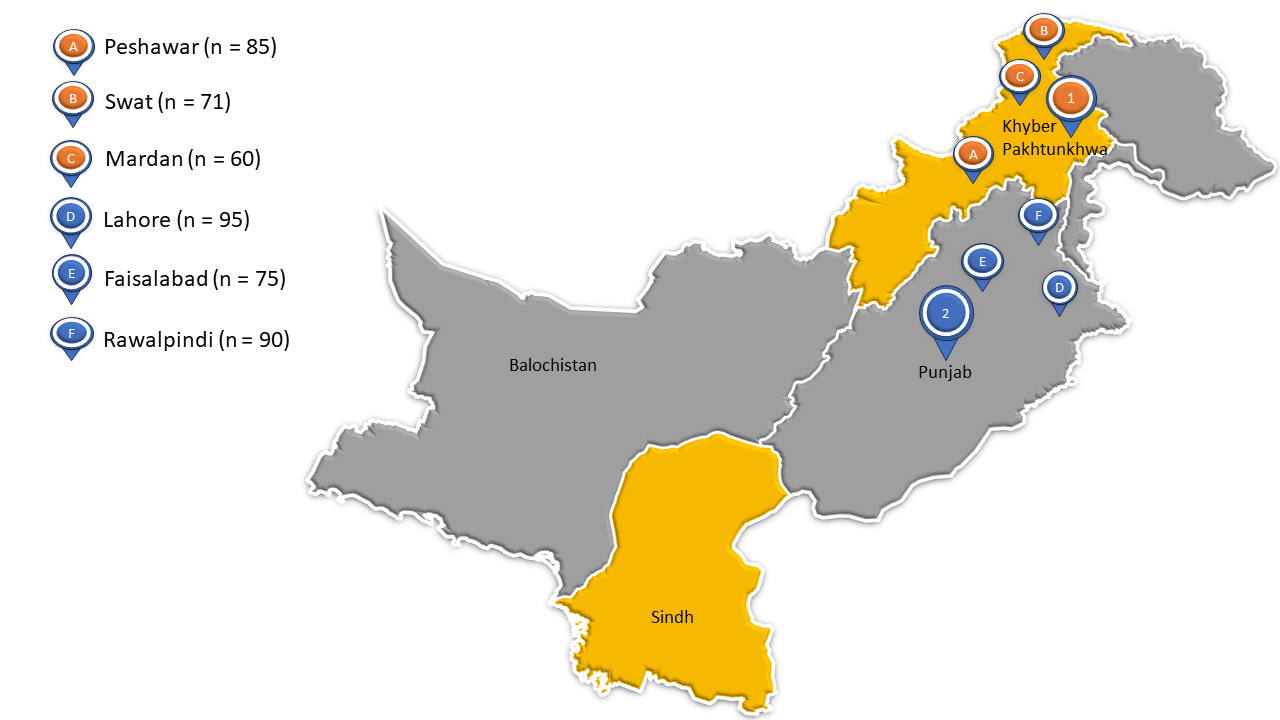


**Supplementary Figure S1: Selected data settings for the current study (“n” shows number of selected participants having history of UTI)**

**
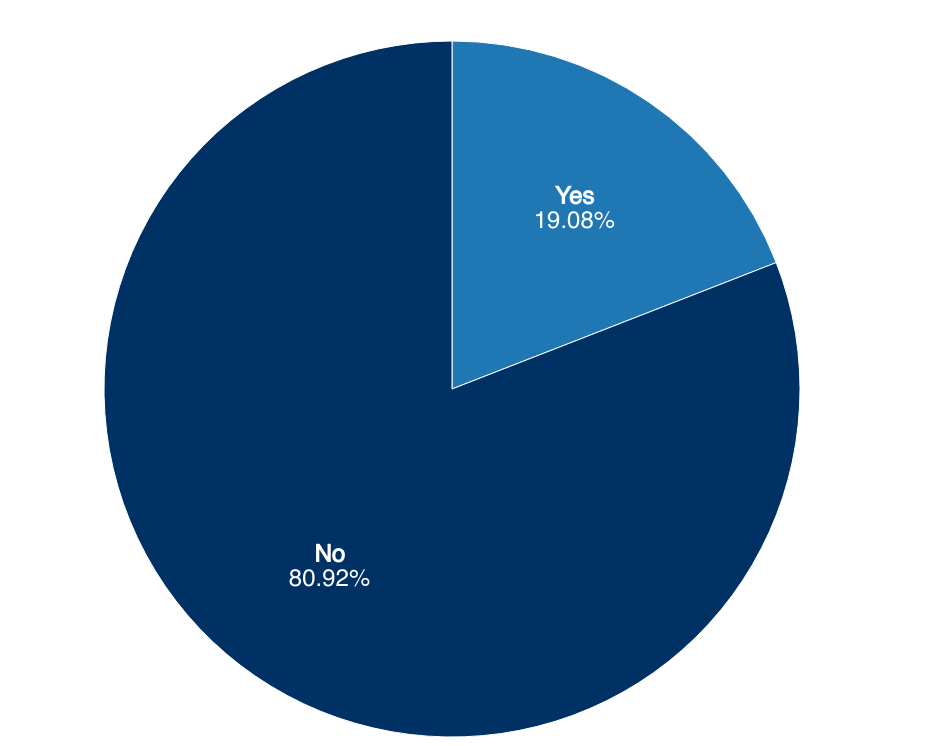
**

**Supplementary Figure S2. Frequency of UTI in the selected population**

**Supplementary Table S2. Association of multi-dimension poverty with treatment-seeking behavior and antibiotic misuse in the selected provinces**

|  | | **Province** | | | | | | | | | |
| --- | --- | --- | --- | --- | --- | --- | --- | --- | --- | --- | --- |
|  |  | **Kpk** | | | | **P-value** | **Punjab** | | | | **P-value** |
|  |  | **Multi-dimension poverty** | | | |  | **Multi-dimension poverty** | | | |  |
|  |  | **ND** | **VD** | **D** | **SD** |  | **ND** | **VD** | **D** | **SD** |  |
| UTI Treated | No | 7 (41.2) | 43 (46.7) | 13 (61.9) | 19 (22.4) | 0.001 | 8 (50.0) | 40 (41.2) | 15 (51.7) | 32 (30.8) | 0.11 |
|  | Yes | 10 (58.8) | 49 (53.3) | 8 (38.1) | 66 (77.6) |  | 8 (50.0) | 57 (58.8) | 14 (48.3) | 72 (69.2) |  |
| Consultation | Physician | 3 (30.0) | 24 (49.0) | 2 (25.0) | 3 (4.5) | <0.001* | 3 (37.5) | 30 (52.6) | 7 (50.0) | 3 (4.2) | <0.001* |
|  | Pharmacist | 6 (60.0) | 20 (40.8) | 2 (25.0) | 13 (19.7) |  | 3 (37.5) | 21 (36.8) | 4 (28.6) | 8 (11.1) |  |
|  | Friends and Family | 1 (10.0) | 4 (8.2) | 4 (50.0) | 48 (72.7) |  | 2 (25.0) | 5 (8.8) | 2 (14.3) | 60 (83.3) |  |
|  | other | 0 (0.0) | 1 (2.0) | 0 (0.0) | 2 (3.0) |  | 0 (0.0) | 1 (1.8) | 1 (7.1) | 1 (1.4) |  |
| Use of antibiotics UTI | No | 3 (23.1) | 13 (21.0) | 4 (33.3) | 12 (15.4) | 0.47* | 2 (20.0) | 19 (25.0) | 3 (17.6) | 9 (11.1) | 0.16* |
|  | Yes | 10 (76.9) | 49 (79.0) | 8 (66.7) | 66 (84.6) |  | 8 (80.0) | 57 (75.0) | 14 (82.4) | 72 (88.9) |  |
| Self-medication | No | 3 (30.0) | 26 (53.1) | 2 (25.0) | 7 (11.5) | <0.001* | 3 (37.5) | 32 (56.1) | 8 (57.1) | 8 (11.3) | <0.001* |
|  | Yes | 7 (70.0) | 23 (46.9) | 6 (75.0) | 54 (88.5) |  | 5 (62.5) | 25 (43.9) | 6 (42.9) | 63 (88.7) |  |
| Antibiotic course completed | No | 1 (10.0) | 10 (20.4) | 4 (50.0) | 52 (85.2) | <0.001* | 0 (0.0) | 10 (17.5) | 3 (21.4) | 63 (88.7) | <0.001* |
|  | Yes | 9 (90.0) | 39 (79.6) | 4 (50.0) | 9 (14.8) |  | 8 (100.0) | 47 (82.5) | 11 (78.6) | 8 (11.3) |  |
| Skip antibiotic dose | No | 7 (70.0) | 28 (73.7) | 3 (75.0) | 7 (70.0) | 0.99* | 5 (62.5) | 36 (73.5) | 8 (72.7) | 5 (62.5) | 0.86* |
|  | Yes | 3 (30.0) | 10 (26.3) | 1 (25.0) | 3 (30.0) |  | 3 (37.5) | 13 (26.5) | 3 (27.3) | 3 (37.5) |  |

Kpk – Khyber Pakhtunkhwa, * Fisher exact test, ND – Not deprived. VD – Vulnerable to deprivation, D – Deprived, SD – Severely deprived
